# Prolonged Life Expectancy for Those Dying of Stroke by Achieving the Daily PM_2.5_ Targets

**DOI:** 10.1002/gch2.202000048

**Published:** 2020-10-13

**Authors:** Zengliang Ruan, Jinlei Qi, Peng Yin, Zhengmin (Min) Qian, Jiangmei Liu, Yunning Liu, Yin Yang, Huan Li, Shiyu Zhang, Steven W. Howard, Hualiang Lin, Lijun Wang

**Affiliations:** ^1^ Department of Epidemiology School of Public Health Sun Yat‐Sen University Guangzhou 510080 China; ^2^ National Center for Chronic and Noncommunicable Disease Control and Prevention Chinese Center for Disease Control and Prevention Beijing 100050 China; ^3^ Department of Epidemiology and Biostatistics College for Public Health & Social Justice Saint Louis University Saint Louis MO 63104 USA; ^4^ Department of Health Management & Policy College for Public Health & Social Justice Saint Louis University Saint Louis MO 63104 USA

**Keywords:** air quality standards, fine particulate matter, life expectancy, multi‐city studies, stroke

## Abstract

This time‐series study collects data on stroke‐related mortality, years of life lost (YLL), air pollution, and meteorological conditions in 96 Chinese cities from 2013 to 2016 and proposes a three‐stage strategy to generate the national and regional estimations of avoidable YLL, gains in life expectancy and stroke‐related population attributable fraction by postulating that the daily fine particulate matter (PM_2.5_) has been kept under certain standards. A total of 1 318 911 stroke deaths are analyzed. Each 10 µg m^−3^ increment in PM_2.5_ at lag_03_ is associated with a city‐mean increase of 0.31 (95% CI: 0.19, 0.44) years of life lost from stroke. A number of 914.11 (95% CI: 538.28, 1288.94) years of city‐mean life lost from stoke could be avoided by attaining the WHO's Air Quality Guidelines (AQG) (25 µg m^−3^). Moreover, by applying the AQG standard, 0.11 (0.08, 0.15) years of life lost might be prevented for each death, and about 0.91% (95% CI: 0.62%, 1.19%) of the total years of life lost from stroke might be explained by the daily excess PM_2.5_ exposure. This study indicates that stroke patients can have a longer life expectancy if stricter PM_2.5_ standards are put in place, especially ischemic stroke patients.

## Graphical abstract

This study aims to examine the relationships between daily fine particulate matter PM_2.5_ pollution and stroke‐related years of life lost YLL and potential gains in life expectancy by achieving different PM_2.5_ standards in 96 Chinese cities from 2013 to 2016. The results show that daily PM_2.5_ exposure is associated with YLL due to stroke, and that life expectancy can be longer by adopting stricter PM_2.5_ standards.

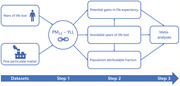

## 1 Introduction

Stroke leads to both high mortality and disability in the world.^[^ ^1^, ^2^ ^]^ The Global Burden of Diseases, Injuries, and Risk Factors Study 2017 (GBD 2017) indicated that stroke caused more than six million life lost worldwide (11.02% of all deaths), which corresponded to 113 million years of life lost (6.89% of the total loss).^[^ ^3^ ^]^ In China, an estimated 106 people per 100 000 population died from stroke in 2017.^[^ ^4^ ^]^

A number of literatures have demonstrated associations between ambient fine particulate matter pollution (PM_2.5_) with premature mortality from stroke.^[^ ^5^ ^]^ Our previous studies also suggested that each 10 µg m^−3^ increase in the moving average (lag_03_) PM_2.5_ was associated with an increase of 3.07% in the stroke mortality and that a reduction in pollution levels during the Asian Games was related to a decreased risk of stroke mortality.^[^ ^6^ ^]^ In addition, findings from the USA also suggested a 1.78% increase in stroke death for each 10 µg m^−3^ greater increment of 2‐day averaged PM_2.5_.^[^ ^7^ ^]^

A few studies used years of life lost (YLL) or excess risk as indicators to examine the disease burden due to air pollution.^[^ ^8^, ^9^ ^]^ For example, Yang et al. reported that a 10 µg m^−3^ increase in 2‐day average PM_10_ concentration was associated with 0.9 YLL in Guangzhou, China.^[^ ^10^ ^]^ One study from another Chinese city also reported that PM_10_ exposure was associated with increases in both stroke mortality and stroke‐related years of life lost.^[^ ^11^ ^]^ However, no previous report has addressed the relation between daily PM_2.5_ exposure and YLL due to stroke.

Considering the widely reported association between PM pollution and increased premature stroke mortality, we hypothesized that daily PM_2.5_ exposure was also associated with increased years of life lost and could therefore lead to shorter life expectancy among those stroke patients. In other words, reducing PM_2.5_ to a certain level could mean prolonged life expectancy for stroke survivors.

This research aimed to estimate the possible attainments in stroke‐related life expectancy by reaching different standards for daily PM_2.5_ level, which included the Chinese National Ambient Air Quality Standard (NAAQS), as well as the AQG and three Interim Targets (ITs) set by WHO, in 96 Chinese cities during the years of 2013 to 2016.

## 2 Methods

### 2.1 Data Collection

This time‐series study was based on a nationwide dataset containing the daily data on stroke mortality, concentration of pollutants, climatic conditions in 96 Chinese cities (Table S1, Supporting Information) from 2013 to 2016. We chose these cities based on the criteria: 1) data on the daily stroke mortality counts, air pollution, and meteorological factors of each city were available and reliably collected during the study period; 2) there were no adjustments to the cities’ administrative area during the study period; and 3) the daily stroke mortality counts in these cities did not have large fluctuations during the study period.

**Figure 1:**
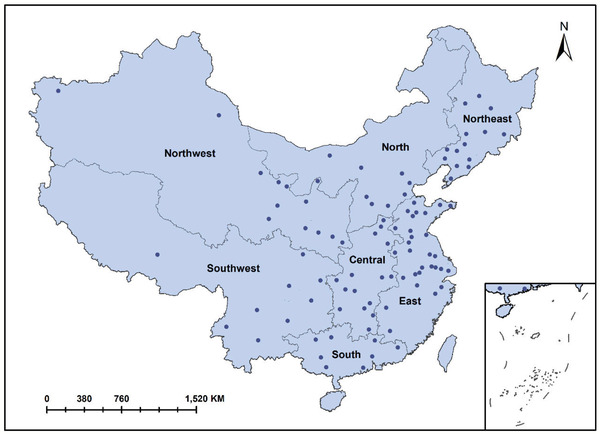
Location of the 96 cities across seven regions of mainland China. The cities are indicated by dark blue dots (•).

We classified the study cities into seven regions according to previous experience,^[^ ^12^ ^]^ which was based on the cities’ geographical location, custom, culture, and climate (**Figure** **1**): northeast (*n* = 13), north (*n* = 8), northeast (*n* = 14), central (*n* = 15), east (*n* = 29), southwest (*n* = 9) and South (*n* = 8).

The stroke mortality data were extracted from the Cause of Death Reporting System (CDRS), which was managed by the Chinese Center for Disease Control and Prevention (China CDC). The stroke mortality data quality was ensured by multiple administrative levels in the China CDC network. The mortality data have been used to evaluate disease burden and the health impact of air pollution.^[^ ^13^, ^14^ ^]^ The International Classification Disease, tenth revision (ICD‐10), I60–I64 were classified as stroke, which was further divided into hemorrhagic stroke (ICD‐10 code: I60‐I61) and ischemic stroke (ICD‐10 code: I63).

**Figure 2:**
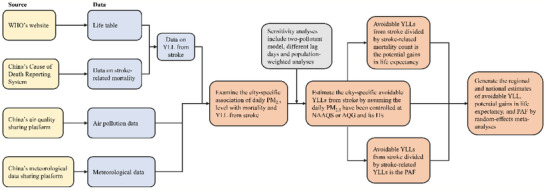
The data sources and statistical analysis process of this study. Abbreviations: WHO = World Health Organization; YLL = years of life lost; PM_2.5_ = particulate matter with an aerodynamic diameter less than or equal to 2.5 µm; NAAQS = Chinese National Ambient Air Quality Standard; AQG = WHO's Air Quality Guidelines; ITs = Interim Targets; PAF = population attributable fraction.

Additionally, we obtained the Chinese national life table (2013‐2016) from the WHO's website (http://apps.who.int/gho/data/node.main.687?lang = en). We matched age and sex of each death to the Chinese national life table, then calculated their corresponding YLL, following the model established in a previous study, the R codes for calculating the YLL were provided in the Supplemental material.^[^ ^15^ ^]^ We then summed the overall YLLs for all stroke daily deaths in a city, and this value represented the daily YLLs from stroke of the cities. The flow chart of data sources and the statistical analysis was summarized in **Figure** **2**.

### 2.2 Air Pollutants and Meteorological Factors

The real‐time concentrations of PM_2.5_, sulfur dioxide (SO_2_), nitrogen dioxide (NO_2_), and ozone (O_3_) were measured by state‐controlled monitoring stations,^[^ ^16^ ^]^ which were available from the air quality sharing platform of China (http://106.37.208.233:20035). We then averaged the daily PM_2.5_, SO_2_, NO_2_ and 8‐h maximum O_3_ concentrations for each city.

Furthermore, the daily relative humidity (%) and temperature (°C) data were downloaded from the open website of China's meteorological data sharing platform (http://data.cma.cn).

### 2.3 Statistical Analysis

#### 2.3.1 Descriptive Analyses

The daily mean concentrations of air pollution, meteorological factors, stroke mortality, and YLL were summarized using a descriptive analysis. The Spearman rank correlation was employed to examine the correlations between pollutants and meteorological factors.

#### 2.3.2 Step One: City‐Specific Associations between PM_2.5_ and Stroke‐Related Mortality and YLL

The associations of daily PM_2.5_ with stroke‐related outcomes were examined through a three‐stage strategy at both the national and regional levels, and similar approach has been recently introduced in our previous studies on all‐cause mortality.^[^ ^12^, ^17^ ^]^ The city‐specific relation between PM_2.5_ level and stroke‐related death count were first analyzed using a generalized additive model (GAM) with a quasi‐Poisson link. The association between stroke‐related YLL and PM_2.5_ was examined by a GAM with Gaussian link. In this stage, daily mortality count or YLL from stroke in each city were the dependent variables with daily mean PM_2.5_ concentration as the independent variable. Both day of the week (DOW) and public holidays (PH) were adjusted in the analyses. Temperature, relative humidity, long‐term and seasonal trends were controlled by the penalized smoothing splines function.^[^ ^18^ ^]^ The supplemental Table S2, Supporting Information display a list of covariates and degrees of freedom (df) of the smoothers being used in the models, which were selected based on previously similar studies.^[^ ^19^ ^]^ A df of six per year for temporal trends, a df of six for temperature, and a df of three for relative humidity were used to adjusted for the potential nonlinear relationships. The formula can be specified as:

(1) $$\begin{array}{c} \begin{array}{l} \text{YLL}\;\;=\;\;\alpha +\beta^{*}{\text{PM}}_{2.5}+\beta_{1}{}^{*}\;\text{DOW}+\beta_{2}{}^{*}\;\text{PH}+s\left(t,df=6\text{/year}\right)\\ +s\left(\text{TM},df=6\right)+s\left(\text{RH},df=3\right) \end{array} \end{array}$$

In this formula, YLL means the years of life lost from stroke mortality, PM_2.5_ is the concentration of fine particulate matter, TM is air temperature, and RH is relative humidity.

We evaluated the lagged effects of daily PM_2.5_ on stroke‐related mortality and YLL for the current day (lag_0_), 1 day (lag_1_), 2 days (lag_2_), 3 days (lag_3_) before, and moving averages for the current day and the previous 1, 2, and 3 days (lag_01_, lag_02_, lag_03_). We also performed these analyses for two main types of strokes: ischemic and hemorrhagic strokes.

#### 2.3.3 Step Two: Calculate the City‐Specific Avoidable YLLs

At the second stage, based on the established model, we evaluated the city‐specific avoidable YLLs from stroke by postulating that the daily PM_2.5_ level had been kept under a series of air pollution standards, such as the NAAQS and AQG. Then, we calculated the possible attainments in stroke‐related life expectancy by averaging the avoidable YLLs on the city‐specific stroke mortality; this calculation process can be expressed by the following formula:

(2) $$\begin{array}{c} \begin{array}{l} \text{Potential}\;\;\text{benefits}\;\;\text{to}\;\;\text{life}\;\;\\ \text{expectancy}\;\;=\;\;\frac{\text{Avoidable}\;\;\text{stroke}-\text{related}\;\;\text{YLLs}}{\text{Stroke}\;\;\text{mortality}\;\;\text{count}} \end{array} \end{array}$$

In this formula, the avoidable stroke‐related YLLs was the estimated years of life lost related to stroke that can be avoided if daily PM_2.5_ level was kept under different standards in each city. The stroke mortality count refers to the overall death number due to stroke of the corresponding city. The reference standards being used for PM_2.5_ level in our study included the AQG (25 µg m^−3^) and IT‐1 (75 µg m^−3^, also the currently used NAAQS), IT‐2 (50 µg m^−3^), and IT‐3 (37.5 µg m^−3^).

In order to show the proportion of stroke‐related YLL caused by a higher‐than‐standard level of PM_2.5_ exposure, we computed the city‐specific population attributable fraction (PAF) by the formula as below:

(3) $$\begin{array}{c} \text{PAF}\;\;=\;\;\frac{\text{Avoidable}\;\;\text{stroke}-\text{related}\;\;\text{YLLs}}{\text{Stroke}\;\;-\;\;\text{related}\;\;\text{YLLs}} \end{array}$$

Where PAF was the population attributable fraction; avoidable stroke‐related YLLs was the estimated years of life lost from stroke that can be saved if the daily PM_2.5_ level was kept under different standards in the study city; and stroke‐related YLL is the total YLL for stroke‐related deaths of the corresponding city.

#### 2.3.4 Step Three: Calculating the Overall Potential Life Expectancy Benefits and PAF

At the third stage, we separately pooled the values of different cities to generate the regional and national estimates of stroke‐YLL association, stroke‐related avoidable YLL, potential life expectancy benefits and the population attributable fraction by conducting random‐effect meta‐analyses.^[^ ^20^, ^21^ ^]^

#### 2.3.5 Sensitivity Analyses

To verify the robustness of the results, a number of sensitivity analyses were conducted. First, we tested two‐pollutant models by simultaneously including PM_2.5_ and one other pollutant (e.g., SO_2_, NO_2_ or O_3_) in the model analyses. We also employed the mixed‐effects generalized additive models to estimate the national and regional associations, with the city variable being used as a random‐effect term. Furthermore, the city‐specific coefficients for the associations of daily PM_2.5_ with stroke‐related mortality count and YLL were statistically weighted to represent five million Chinese population to conduct the population‐weighted meta‐analyses. Finally, we used a meta‐regression method to check whether the differential associations across the cities might be attributable to the following city characteristics: 1) annual mean PM_2.5_, CO, O_3_, NO_2_ and SO_2_ levels; 2) geographical features and annual meteorological conditions such as elevation, temperature, precipitation, relative humidity, and air pressure; 3) socio‐demographic characteristics such as education, poverty, population density, gross domestic product (GDP) and per capita GDP.

The data analyses were conducted with the “mgcv” and “metafor” packages in R software (version 3.6.2).^[^ ^21^, ^22^ ^]^ Two‐sided tests with *p*‐values less than 0.05 were considered statistically significant for the analyses. And the sample R codes for our main analyses were illustrated in the supplemental material.

### 2.4 Data Availability

The Chinese national life table (2013–2016) are publicly available at the WHO's website (http://apps.who.int/gho/data/node.main.687?lang=en). The stroke mortality data were extracted from the CDRS that was managed by the China CDC. The real‐time concentrations of air pollutants were available from the air quality sharing platform of China (http://106.37.208.233:20035), and the meteorological data were downloaded from the open website of China's meteorological data sharing platform (http://data.cma.cn).

## 3 Results

### 3.1 Descriptive Results

A total of 1318911 stroke deaths were collected. Among them, 744 682 were males, and 574 229 were females. They included 567 787 deaths due to ischemic strokes, 668 343 deaths due to hemorrhagic strokes, and 82 781 undetermined strokes. The number of cities, pollution levels, meteorological factors, daily mean mortality count, and YLL from stroke in different regions are displayed in **Table** **1**. The daily PM_2.5_, SO_2_, NO_2_ and O_3_ levels were 46.92 to 87.24, 26.13 to 59.53, 28.02 to 45.21, and 71.19 to 96.20, respectively. The daily mean temperature ranged from 8.57 to 22.01 °C, while the daily mean relative humidity ranged from 52.11% to 78.28%. In addition, the daily mortality counts from stroke (ischemic and hemorrhagic stroke) ranged from 7.25 (3.61 and 4.83) to 15.06 (7.86 and 9.10), and the corresponding daily mean YLLs ranged from 97.72 (37.06 and 69.69) to 218.20 (93.89 and 126.13).

**Table 1:** Characteristics of the study cities by regions, 2013–2016

Variable	Northwest	North	Northeast	Central	East	Southwest		South	National
Number of cities	13	8	14	15	29	9		8	96
Daily mean concentration of air pollutants [µg m^−3^]									
PM_2.5_	54.12	87.24	58.20	76.12	72.66	46.92	49.90		66.08
SO_2_	34.26	59.53	41.35	33.00	42.68	27.20	26.13		38.80
NO_2_	29.96	45.21	35.37	35.81	38.56	28.02	30.47		35.68
O_3_	80.93	96.20	86.35	81.20	89.56	71.19	87.24		85.60
Daily mean meteorological factors									
Temperature [°C]	11.53	12.36	8.57	17.33	15.92	17.14	22.01		14.88
Relative humidity [%]	52.11	55.36	61.58	73.33	71.30	66.70	78.28		66.72
Daily mean stroke‐related mortality count									
Total stroke	7.25	12.17	15.06	11.27	14.03	14.20	9.25		12.52
Ischemic stroke	4.04	6.36	7.86	4.43	7.02	5.09	3.61		6.01
Hemorrhagic stroke	4.83	6.15	7.35	6.59	6.71	9.10	4.85		6.63
Daily mean stroke‐related YLL [years]									
Total stroke	97.72	157.80	218.20	145.22	160.33	178.93	114.24		158.29
Ischemic stroke	45.50	67.60	93.89	48.16	67.57	53.23	37.06		63.45
Hemorrhagic stroke	71.94	94.89	126.13	94.84	90.69	124.46	69.69		96.96

Abbreviations: PM_2.5_ = particulate matter with an aerodynamic diameter less than or equal to 2.5 µm; SO_2_ = sulfur dioxide; NO_2_ = nitrogen dioxide; O_3_ = ozone; YLL = years of life lost.

Low to moderate correlations were observed between the pollutants and meteorological factors (Table S3, Supporting Information). For example, PM_2.5_ was shown to have relatively low and positive relation with O_3_ and SO_2_ (coefficients: 0.36 and 0.29, respectively) and was shown to have a moderate positive correlation with NO_2_ with a coefficient of 0.49. PM_2.5_ also had very low negative correlations with relative humidity and air temperature, and the coefficients were −0.01 and −0.15, respectively.

### 3.2 The Relation between PM_2.5_ Level and Stroke‐Related YLL

**Figure 3:**
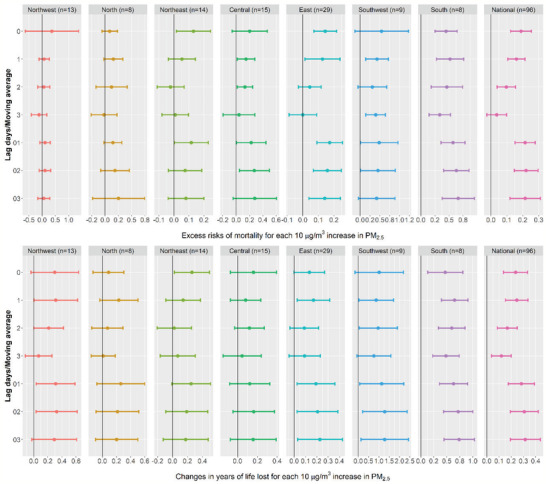
Associations of ambient PM_2.5_ with mortality and YLL. Moving average of lag 0 to lag 3 (lag_03_) for daily PM_2.5_ level was used. Abbreviations: PM_2.5_ = particulate matter with an aerodynamic diameter less than or equal to 2.5 µm; YLL = years of life lost.

The regional and lag‐specific relation between daily mean PM_2.5_ level and stroke‐related mortality and YLL varied in single‐pollutant models (**Figure** **3**). We observed that the daily PM_2.5_ concentrations were positively associated with stroke‐related excess risk of mortality in all the study regions, and the daily PM_2.5_ concentrations were also positively associated with YLL from stroke in the seven regions and the national level, with the lag_03_ models being the strongest lag association. Each 10 µg m^−3^ increment in the daily PM_2.5_ levels at lag_03_ was related to an excess mortality risk of 0.22 (95% CI: 0.12, 0.31) and an increase of 0.31 (95% CI: 0.19, 0.44) years of life lost from stroke at the national level (Table S4, Supporting Information), though the associations varied by region according to the region‐specific analyses. For example, the South region had the highest association between daily PM_2.5_ level and stroke‐related mortality count, with each 10 µg m^−3^ increment in the daily PM_2.5_ levels at lag_03_ was related to an excess risk of 0.66 (95% CI: 0.38, 0.95). The Northwest region, on the other hand, had the lowest excess risk of 0.06 (95% CI: −0.16, 0.29) and was not statistically significant. The Southwest region had the highest correlation between PM_2.5_ and stroke‐related YLL with the coefficient of 1.28 (95% CI: 0.14, 2.43), and the Central area had the lowest correlation (β = 0.16, 95% CI: −0.07, 0.39). A series of model diagnostic graphs such as residual plots (Figure S1, Supporting Information), plots of partial autocorrelation function (PACF, Figure S2, Supporting Information), and *Q*–*Q* plots (Figure S3, Supporting Information) for six provincial capital cities were provided as the online supplemental figures. These results suggested that the goodness of fit of the models were acceptable.

The sensitivity analyses showed consistent associations of PM_2.5_ with daily mortality count and YLL from stroke (Table S4, Supporting Information). For example, each 10 µg m^−3^ increment in the daily PM_2.5_ level may cause excess risks of 0.19 (95% CI: 0.10, 0.29), 0.14 (95% CI: 0.06, 0.22), or 0.20 (95% CI: 0.11, 0.30) in mortality from stroke at the national level when adjusting for SO_2_, NO_2_, or O_3_, respectively. Moreover, we also observed increments of 0.31 (95% CI: 0.18, 0.43), 0.28 (95% CI: 0.16, 0.41), or 0.32 (95% CI: 0.19, 0.44) in stroke‐related YLL per 10 µg m^−3^ increase in the daily PM_2.5_ level after adjusting for SO_2_, NO_2_, or O_3_, respectively. We observed similar trend of associations between PM_2.5_ with YLL in the mix‐effect analysis, with a coefficient of 0.18 (95% CI: 0.09, 0.27) for YLL from stroke at the national level (Table S5, Supporting Information). Additionally, in our population‐weighted analyses, we observed larger effect estimates, and each 10 µg m^−3^ increment in the daily PM_2.5_ level is related to an excess risk of 0.32 (95% CI: 0.15, 0.49) and an increment of 0.95 (95% CI: 0.33, 1.57) in stroke‐related YLL (Table S6, Supporting Information).

### 3.3 Avoidable Stroke‐related YLL, Potential Gains in Life Expectancy

**Table** **2** shows the avoidable YLL and PAF. Stricter air pollution standards could generally result in reductions of stroke‐related years of life lost, and we estimated that a relatively low city‐mean of 10.89 (95% CI: 2.44, 19.33) years of life lost from stoke may be saved by achieving the current daily standard (75 µg m^−3^), which could increase to 914.11 YLL (95% CI: 539.28, 1288.94) by attaining the WHO's AQG (25 µg m^−3^). Moreover, different effect estimates were found in the seven regions. For example, the Southwest region had the largest, significant city‐mean avoidable stroke‐related YLL of 3422.37 (95% CI: −1202.10, 8046.85) that could be attained by achieving the WHO's AQG, while the Northwest region only yield 411.57 YLL avoided (95% CI: −164.68, 987.83) under the same standard.

**Table 2:** The avoidable stroke‐related years of life lost, potential gains in life expectancy and PAF for those dying of stroke by enhancing PM_2.5_ level to Chinese and WHO's guidelines in the study cities during 2013–2016

Region	Avoidable YLL (95% CI)		Benefits in life expectancy (95% CI)		PAF ([%] 95% CI)	
	China's standard (IT‐1)	WHO's AQG	China's standard (IT‐1)	WHO's AGQ	China's standard (IT‐1)	WHO's AQG
Northwest	17.07 (−7.29, 41.44)	411.57 (−164.68, 987.83)	0.01 (−0.01, 0.04)	0.16 (0.03, 0.29)	0.09 (−0.10, 0.28)	1.15 (0.13, 2.17)
North	295.76 (−135.75, 727.27)	1009.35 (−410.39, 2429.10)	0.02 (−0.01, 0.05)	0.09 (−0.02, 0.19)	0.14 (−0.07, 0.36)	0.64 (−0.17, 1.46)
Northeast	157.58 (−49.10, 364.26)	896.82 (−273.18, 2066.82)	0.02 (0.01, 0.03)	0.07 (−0.003, 0.14)	0.15 (0.05, 0.24)	0.47 (−0.01, 0.95)
Central	46.61 (−90.13, 183.35)	607.45 (−315.24, 1530.13)	0.01 (−0.01, 0.03)	0.08 (−0.01, 0.18)	0.09 (−0.08, 0.27)	0.69 (−0.09, 1.48)
East	5.79 (−11.13, 22.72)	1085.18 (148.42, 2021.94)	0.02 (0.005, 0.03)	0.10 (0.03, 0.17)	0.15 (0.04, 0.26)	0.91 (0.34, 1.48)
Southwest	3.23 (−1.27, 7.74)	3422.37 (−1202.10, 8046.85)	0.0004 (−0.0002, 0.001)	0.18 (0.04, 0.32)	0.19 (−0.10, 0.48)	1.46 (0.36, 2.55)
South	113.33 (5.18, 221.48)	2042.97 (1041.70, 3044.23)	0.04 (0.01, 0.07)	0.17 (0.10, 0.25)	0.33 (0.09, 0.58)	1.46 (0.85, 2.08)
National	10.89 (2.44, 19.33)	914.11 (539.28, 1288.94)	0.001 (0.0001, 0.0014)	0.11 (0.08, 0.15)	0.01 (0.006, 0.01)	0.91 (0.62, 1.19)

Moving average of lag 0 to lag 3 (lag_03_) for daily PM_2.5_ level was used; the reference PM_2.5_ standards were the IT‐1 or Chinese NAAQS (75 µg m^−3^) and WHO's AQG (25 µg m^−3^); abbreviations: PM_2.5_ = particulate matter with an aerodynamic diameter less than or equal to 2.5 µm; YLL = years of life lost; PAF = population attributable fraction; IT = interim targets; NAAQS = National Ambient Air Quality Standards; AQG = Ambient Air Quality Guidelines.

**Figure 4:**
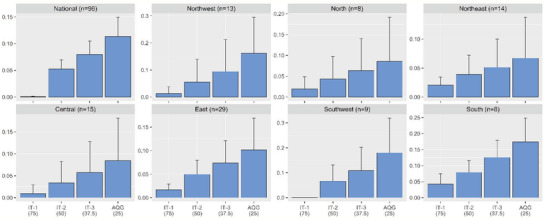
Life gains by achieving different recommend levels for PM_2.5_ control. Moving average of lag 0 to lag 3 (lag_03_) for daily PM_2.5_ level was used. Abbreviations: PM_2.5_ = particulate matter with an aerodynamic diameter less than or equal to 2.5 µm; IT = Interim Targets; AQG = WHO's Ambient Air Quality Guidelines

**Figure** **4** displays the potential gains in life expectancy under different standards in both the regional and national level. Specifically, we found that about 0.001 (95% CI: 0.0001, 0.0014) and 0.11 (95% CI: 0.08, 0.15) years might be saved for each stroke‐related death by achieving China's NAAQS (75 µg m^−3^) and the WHO's AQG (25 µg m^−3^), respectively. Additionally, the largest and smallest significant values of 0.18 (95% CI: 0.04, 0.32) and 0.07 (95% CI: −0.003, 0.14) was estimated in the Southwest and the Northeast regions, respectively, when adopting AQG as the recommended target.

Using NAAQS and AQG as references, we estimated that about 0.01% (95% CI: 0.006%, 0.01%) and 0.91% (95% CI: 0.62%, 1.19%) of the total years of life lost from stroke, respectively, might be explained by the daily excess PM_2.5_ exposures (Table 2). Moreover, the estimates were also found to be heterogeneous among different regions, with the largest significant PAF being found in the South and Southwest region [1.46% (0.85%, 2.08%) and 1.46% (0.36%, 2.55%)] and the smallest being found in the Northeast region [0.47% (−0.01%, 0.95%)].

The stratified analyses for ischemic and hemorrhagic stroke suggested that the benefits in life expectancy of reducing daily PM_2.5_ level were more pronounced for ischemic than hemorrhagic stroke (Tables S7 and S8, Supporting Information). To be specific, we found that an increase of 0.13 (95% CI: 0.08, 0.18) years of life expectancy might be obtained by attaining the AQG (25 µg m^−3^), with a PAF of 1.21% (95% CI: 0.73%, 1.70%) for the total YLLs due to ischemic stroke, while for hemorrhagic stroke, the corresponding benefit in life expectancy was 0.07 (95% CI: 0.02, 0.12) years and a 0.50% (95% CI: 0.18%, 0.82%) PAF for YLL.

Our meta‐regression analysis suggested that the correlation between daily PM_2.5_ level and stroke‐related life expectancy was relatively higher in cities with higher GDP, lower annual O_3_ levels, and higher annual mean temperature (Table S9, Supporting Information). Each IQR (218.20 billion, CNY) increase in average GDP was associated with a 0.18 (0.06, 0.30) increment in the regression coefficient, each IQR (20.45 µg m^−3^) increment in annual mean levels of O_3_ was related to 0.26 (0.01, 0.51) decrease in the regression coefficient, and each IQR (6.15 °C) increment in annual mean temperature was related to 0.39 (0.01, 0.77) increase in the regression coefficient. However, no significant effect was found for other city‐level factors, such as GDP per capita, population density, or elevation.

## 4 Discussion

To our knowledge, this study is among the first efforts to examine the effects of daily air pollution on life expectancy due to stroke mortality. Based on a large dataset covering 96 cities across the seven geographic regions of China, our study suggested that daily PM_2.5_ exposure was associated with stroke‐related years of life lost, especially for ischemic stroke. Moreover, our results suggested that by attaining stricter ambient air pollution standards, a person can have a longer life expectancy.

The observed correlation between daily PM_2.5_ level and stroke‐related life expectancy was supported by the widely‐reported associations between daily PM_2.5_ exposure and premature mortality from stroke. For example, one of our former studies revealed that each 10 µg m^−3^ increase in 4‐day moving average (lag_03_) of daily PM_2.5_ concentration was associated with an excess risk of 3.07% for overall stroke mortality in six Chinese subtropical cities, and about 5.57% of the stroke mortality in this study population was suggested to be attributable to PM_2.5_ exposure levels higher than the WHO's guideline.^[^ ^23^ ^]^ One case‐crossover study conducted in the US found positive associations between PM exposure and ischemic stroke, with an odds ratio of 1.26 per interquartile range increase of the PM_10_ concentration at lag_03_.^[^ ^24^ ^]^ These associations were further supported by a prospective cohort study which suggested a hazard ratio of 1.21 for ischemic stroke per 10 µg m^−3^ increase in the PM_2.5_ concentration.^[^ ^25^ ^]^

Our findings of the possible attainments in life expectancy by achieving daily PM_2.5_ guidelines are in agreement with several other studies. For example, one US study found that an increase of 2.8 µg m^−3^ in annual average PM_2.5_ concentrations was associated with life expectancy losses of 0.15 years in females and 0.13 years in males.^[^ ^26^ ^]^ Results from another study showed that an increase of 2 µg m^−3^ in the annual PM_2.5_ concentration led to a loss of 0.64 years of life in certain areas of Spain.^[^ ^27^ ^]^ Moreover, there is evidence that reducing PM_2.5_ concentrations would substantially increase life expectancy; specifically, one study found that, in the Medicare population, that 23.5% of people would die before the age of 76 years if they were exposed to an annual average PM_2.5_ concentration of 12 µg m^−3^ and that that percentage would decline to 20.1% for a lower an annual average PM_2.5_ level of 7.5 µg m^−3^.^[^ ^28^ ^]^

The linkage between exposure to PM_2.5_ and life expectancy is biologically plausible. The direct effects of PM_2.5_ on lung receptors and the cardiovascular system may lead to acute cardiovascular responses and contribute to cardiovascular dysfunction.^[^ ^29^ ^]^ Another potential mechanism may be the abnormal activation of the hemostatic system.^[^ ^30^ ^]^ Moreover, lung and systemic inflammation, oxidative stress, and the formation of atherosclerotic plaque resulting from PM_2.5_ inhalation may also lead to increased mortality and morbidity from cardiovascular and cerebrovascular diseases.^[^ ^29^, ^31^, ^32^ ^]^ Additionally, evidence from an intervention study showed a significant improvement on the microvascular function in the elderly after a reduction of particulate matter levels via an indoor air filtration system.^[^ ^33^ ^]^

Since 2013, the toughest‐ever clean air policy was implemented in China, which has introduced remarkable improvements in air quality, and significant declines in PM_2.5_ levels have been achieved nationwide.^[^ ^34^ ^]^ Our study provides substantial evidence for the fact that stricter PM_2.5_ standards may lead to longer stroke‐related life expectancy. A relatively larger benefit in life expectancy was observed when adopting AQG (25 µg m^−3^) as the recommended level, compared to the benefits gained by adopting NAAQS (75 µg m^−3^). The model suggested that a lower air quality limit like the WHO's AQG would result in more benefits in life expectancy. The results of this study indicated that 0.11 years of life per stroke survivor would be prolonged in China by attaining the WHO's air quality guideline on daily concentration of PM_2.5_, while the benefits in stroke‐related life expectancy by attainment of the current China's PM_2.5_ NAAQS (75 µg m^−3^) only yield an extremely low value of 0.001 years, which suggested that this standard may be no longer enough in reducing the risk of death from stroke. This also concurs with previous studies which showed that the short‐term PM_2.5_ effects were smaller than the long‐term effects.^[^ ^35^ ^]^ This finding can be partially explained by the cumulative effects of long‐term exposures.^[^ ^36^ ^]^ Nevertheless, study on the short‐term effects of PM_2.5_ may also help us to understand if even a short‐term exposure to higher levels of PM_2.5_ have adverse impacts on life expectancy.

In our stratified analyses, we observed more pronounced inverse associations between PM_2.5_ and YLL from ischemic than from hemorrhagic stroke. These results were consistent with earlier studies that differentiated the impacts of air pollution on ischemic and hemorrhagic stroke. For example, one study concluded that an interquartile range increase in PM_2.5_ may result in a 1.0% increase in ischemic stroke admissions at lag_3_ days in 26 Chinese cities, but no significant association was observed for hemorrhagic stroke.^[^ ^37^ ^]^ In another study, the odds ratios of hospital admission due to ischemic and hemorrhagic strokes were 2.071 and 1.941, respectively, per interquartile range increase in the same‐day PM_2.5_ concentrations.^[^ ^38^ ^]^ One recent long‐term study also found that, for each 10 µg m^−3^ increase in PM_2.5_ concentration, the incident ischemic stroke increased by 20%, which was higher than the increment of 12% found for hemorrhagic stroke.^[^ ^39^ ^]^ Moreover, these findings were further supported by a study which demonstrated that the relative risk was 1.010 for ischemic stroke for each 10 µg m^−3^ increase in PM_2.5_ and 1.004 for hemorrhagic stroke.^[^ ^40^ ^]^

Our sensitivity analyses suggested that the estimated associations were robust. Specifically, results in the two‐pollutant models which further adjusted for several pollutants, including SO_2_, NO_2_, and O_3_, were in accord with those results in the main models, suggesting that these air pollutants were not confounders in the associations between PM_2.5_ and stroke‐related YLL. However, results from the two‐pollutant analyses generally showed the smallest correlations between PM_2.5_ and stroke‐related YLL when we included NO_2_. The reason for this is not clear, but it may because PM_2.5_ and NO_2_ had a moderate positive correlation (*r* = 0.49). Another possible explanation for this might be due to the similar emission sources for these two air pollutants.^[^ ^41^, ^42^ ^]^

The heterogeneous extents in the correlation between daily PM_2.5_ and stroke‐related YLL among the seven regions is consistent with previous reports,^[^ ^16^ ^]^ and the Southwest, South, Northwest and East regions displayed relatively more reliable associations between PM_2.5_ and stroke‐related YLL. However, although the North, Northeast and Central regions also displayed a positive association, they were statistically insignificant. Such heterogeneity might be explained in part by the different chemical constituents of PM_2.5_ in these regions.^[^ ^43^ ^]^ For example, it is reasonable that the PM_2.5_ in the Southwest is more hazardous than that in other regions because much of the particle matter is related to biomass combustion in these two regions, which has been proven to be more toxic than other sources, according to previous reports.^[^ ^43^ ^]^ Recent findings reported by Want et al. (2019) showed that the elemental carbon and some metallic constituents of PM_2.5_ may lead to ischemic stroke.^[^ ^44^ ^]^ Our meta‐regression analysis found that the observed PM_2.5_–YLL association was relatively higher in cities with lower annual O_3_ levels, which might be because people from high polluted environment usually have more awareness of better protective measures.^[^ ^16^ ^]^ Additionally, the areas with higher annual temperature also tended to have higher PM_2.5_–YLL associations, which indicates that this association may be influenced by meteorological factors.

Although based on a large dataset, this study was subject to a few limitations. First, we cannot make a causal conclusion for the relationship between PM_2.5_ and reduced life expectancy due to the time‐series design, which lacked the measurements at the individual level, such as the personal exposure and smoking status which was an established risk factor for stroke.^[^ ^45^ ^]^ In addition, exposure misclassification was likely due to the usage of city‐averaged PM_2.5_ concentrations as a surrogate of exposure. However, in such a large epidemiologic study, it is difficult to directly measure individual exposures, and city‐averaged PM_2.5_ levels have been widely adopted in previous studies.^[^ ^46^, ^47^ ^]^ Another potential problem of this study was that our findings may be somewhat limited by the representativeness of some geographical regions which included relatively fewer cities, especially the North and South regions. Moreover, the collinearity between PM_2.5_ and other air pollutants may also be a weakness, but the robustness of our findings was further confirmed in the two‐pollutant models. Despite these limitations, this research certainly adds to our understanding of the benefits of PM_2.5_ control.

## 5 Conclusion

This nationwide study suggests that daily PM_2.5_ exposure is associated with YLL due to stroke, and that life expectancy can be longer by reducing daily PM_2.5_ concentrations, especially for ischemic stroke. Our results provide further evidence for the fact that the current PM_2.5_ standard may not be enough in protecting population's mortality risk from stroke, and future prospective studies on this topic will need to be undertaken.

## 6 Experimental Section

The study was approved by the Ethical Review Committee of the School of Public Health, Sun Yat‐Sen University ([2019] No.149). As all of the analyses were based on aggregated data, this study did not require individual consent. The Strengthening the Reporting of Observational Studies in Epidemiology (STROBE) guidelines were followed in reporting the study.

## Conflict of Interest

The authors declare no conflict of interest.

## Supporting information

Supporting Information
