# Supplementary material for: Prolonged Life Expectancy for Those Dying of Stroke by Achieving the Daily PM2.5 Targets
Source: Glob Chall. 2020 Oct 13;4(12):2000048. doi: 10.1002/gch2.202000048 (PMC7713556; doi:10.1002/gch2.202000048)
Supplement: Supplementary file 1 — Supporting Information [file GCH2-4-2000048-s001.pdf]

© 2020 Wiley-VCH GmbH

Global Challenges
Open Access

Supporting Information

for *Global Challenges*, DOI: 10.1002/gch2.202000048

Prolonged Life Expectancy for Those Dying of Stroke by Achieving the Daily $PM_{2.5}$ Targets

Zengliang Ruan, Jinlei Qi, Peng Yin, Zhengmin (Min) Qian, Jiangmei Liu, Yunning Liu, Yin Yang, Huan Li, Shiyu Zhang, Steven W. Howard, Hualiang Lin, and Lijun Wang**

Supporting Information

Prolonged Life Expectancy for Those Dying of Stroke by Achieving the Daily $PM_{2.5}$ Targets

Zengliang Ruan, Jinlei Qi, Peng Yin, Zhengmin (Min) Qian, Jiangmei Liu, Yunning Liu, Yin Yang, Huan Li, Shiyu Zhang, Steven W. Howard, Hualiang Lin, Lijun Wang**

Glossary: $PM_{2.5}$ = fine particulate matter; YLL = years of life lost; GAM = generalized additive model; CI = confidence interval; WHO = World Health Organization; AQG = Air Quality Guidelines; GBD = Global Burden of Diseases; NAAQS = Chinese National Ambient Air Quality Standard; IT = interim target; CDRS = Cause of Death Reporting System; CDC = center for disease control and prevention; ICD-10 = The International Classification Disease, 10th revision; SO_2 = sulfur dioxide; NO_2 = nitrogen dioxide; O_3 = ozone; DOW = day of the week; PH = public holidays; df = degrees of freedom; TM = air temperature; RH = relative humidity; PAF = population attributable fraction; GDP = Gross Domestic Product; IQR = interquartile range; ACF = autocorrelation function

Sample R codes

The calculation of years of life lost (YLL).

```
## Import the life expectancy data and sample data on IHD deaths

LifeExpectancy<-read.csv("D:\\LifeTable.csv")

IHDdeaths<-read.csv("D:\\IHDdeaths.csv")

IHDdeaths$deaddate<-as.Date(IHDdeaths$deaddate, "%Y/%m/%d")

IHDdeaths$year<-year(IHDdeaths$deaddate)


## Define the age groups

IHDdeaths$AgeGroup[IHDdeaths$age<1]<-"<1"

IHDdeaths$AgeGroup[IHDdeaths$age>=1 & IHDdeaths$age<5]<-"<4"

IHDdeaths$AgeGroup[IHDdeaths$age>=5 & IHDdeaths$age<10]<-"<9"

IHDdeaths$AgeGroup[IHDdeaths$age>=10 & IHDdeaths$age<15]<-"<14"

IHDdeaths$AgeGroup[IHDdeaths$age>=15 & IHDdeaths$age<20]<-"15-19"

IHDdeaths$AgeGroup[IHDdeaths$age>=20 & IHDdeaths$age<25]<-"20-24"

IHDdeaths$AgeGroup[IHDdeaths$age>=25 & IHDdeaths$age<30]<-"25-29"

IHDdeaths$AgeGroup[IHDdeaths$age>=30 & IHDdeaths$age<35]<-"30-34"

IHDdeaths$AgeGroup[IHDdeaths$age>=35 & IHDdeaths$age<40]<-"35-39"

IHDdeaths$AgeGroup[IHDdeaths$age>=40 & IHDdeaths$age<45]<-"40-44"

IHDdeaths$AgeGroup[IHDdeaths$age>=45 & IHDdeaths$age<50]<-"45-49"

IHDdeaths$AgeGroup[IHDdeaths$age>=50 & IHDdeaths$age<55]<-"50-54"

IHDdeaths$AgeGroup[IHDdeaths$age>=55 & IHDdeaths$age<60]<-"55-59"
```

```
IHDdeaths$AgeGroup[IHDdeaths$age>=60 & IHDdeaths$age<65]<-"60-64"

IHDdeaths$AgeGroup[IHDdeaths$age>=65 & IHDdeaths$age<70]<-"65-69"

IHDdeaths$AgeGroup[IHDdeaths$age>=70 & IHDdeaths$age<75]<-"70-74"

IHDdeaths$AgeGroup[IHDdeaths$age>=75 & IHDdeaths$age<80]<-"75-79"

IHDdeaths$AgeGroup[IHDdeaths$age>=80 & IHDdeaths$age<85]<-"80-84"

IHDdeaths$AgeGroup[IHDdeaths$age>=85 & IHDdeaths$age<90]<-"85-89"

IHDdeaths$AgeGroup[IHDdeaths$age>=90 & IHDdeaths$age<95]<-"90-94"

IHDdeaths$AgeGroup[IHDdeaths$age>=95 & IHDdeaths$age<100]<-"95-99"

IHDdeaths$AgeGroup[IHDdeaths$age>=100]<-"100+"


## Combine data on death and years of life lost

YLLdata<-join(IHDdeaths, LifeExpectancy, by=c("AgeGroup", "sex", "year"),

              type="left")

names(YLLdata)[9]<-"yll"


## Calculate the total years of life lost by city and date

YLLdata1<-ddply(subset(YLLdata, select=c(city, deaddate, yll)),

                .(city, deaddate), colwise(sum))
```

Table S1. The list of cities.

No.	Province	City	Latitude	Longitude
1	Anhui	Bengbu	33.11037248	117.3285674
2	Anhui	Fuyang	32.91778527	115.7016437
3	Anhui	Suzhou	33.86031277	117.2107086
4	Beijing	Beijing	40.18695288	116.4110932
5	Gansu	Tianshui	34.6512979	105.7411811
6	Hebei	Cangzhou	38.2673799	116.7459782
7	Hebei	Xingtai	37.21268069	114.8194542
8	Heilongjiang	Daqing	46.34721027	124.7011813
9	Heilongjiang	Harbin	45.63756951	127.9569287
10	Heilongjiang	Suihua	46.89517006	126.6458032
11	Henan	Anyang	35.88059171	114.3501897
12	Henan	Xinxiang	35.26499853	114.0915639
13	Henan	Zhengzhou	34.62514792	113.4698881
14	Henan	Zhoukou	33.72607527	114.8780697
15	Jiangsu	Lianyungang	34.53481763	119.1328506
16	Jiangsu	Xuzhou	34.36073168	117.5127239
17	Jilin	Jilin	43.58071226	126.8443023
18	Jilin	Siping	43.57160073	124.4731373
19	Jilin	Yanbian	43.14479774	129.1275139
20	Liaoning	Anshan	40.71819465	123.0076977

21	Liaoning	Benxi	41.23034338	124.5664724
22	Liaoning	Dalian	39.60071735	122.2154122
23	Liaoning	Dandong	40.54791888	124.3959709
24	Liaoning	Fuxin	42.28122402	121.9559376
25	Liaoning	Jinzhou	41.47818111	121.6225153
26	Liaoning	Shenyang	42.09768454	123.1371311
27	Liaoning	Tieling	42.64368007	124.1703741
28	Shaanxi	Baoji	34.37980922	107.2031598
29	Shaanxi	Shangluo	33.65013738	109.8996892
30	Shaanxi	Xian	34.1114387	108.7907707
31	Shandong	Binzhou	37.50287722	117.8302653
32	Shandong	Heze	35.15579967	115.6921261
33	Shandong	Jinan	36.73835363	117.0920564
34	Shandong	Laiwu	36.27698046	117.6597355
35	Shandong	Weifang	36.54039692	119.0769118
36	Shandong	Weihai	37.12465083	121.9813021
37	Shandong	Yantai	37.24631023	120.8073299
38	Shandong	Zaozhuang	34.91917688	117.3952413
39	Shandong	Zibo	36.60944072	118.057108
40	Shanxi	Jinzhong	37.3290385	112.9639443
41	Shanxi	Taiyuan	37.96112892	112.3150466
42	Tianjin	Tianjin	39.31417415	117.3249732

43	Gansu	Jinchang	38.43026804	102.0517784
44	Gansu	Wuwei	38.16606896	103.0520201
45	Gansu	Zhangye	38.95262888	99.85250219
46	Neimentgu	Bayannaoer	41.45278309	107.5781078
47	Neimentgu	Huhehaote	40.59376572	111.4963997
48	Ningxia	Shizuishan	39.01235979	106.5140045
49	Ningxia	Zhongwei	37.04914025	105.4673285
50	Xinjiang	Hami	43.04140931	93.51352336
51	Xinjiang	Kezi	39.70203691	75.98811184
52	Qinghai	Haidong	36.33633386	102.3394999
53	Qinghai	Huangnan	35.02907233	101.6313118
54	Tibet	Lhasa	30.0348938	91.09014949
55	Anhui	Anqing	30.61173105	116.5952459
56	Anhui	Huangshan	29.91097851	118.0764606
57	Anhui	Ma'anshan	31.5366855	118.6212196
58	Anhui	Tongling	30.94267768	117.8851581
59	Anhui	Wuhu	31.07499815	118.3289256
60	Chongqing	Chongqing	30.05968535	107.87553
61	Guangdong	Guangzhou	23.35097248	113.5372608
62	Guangdong	Jiangmen	22.2820937	112.6742817
63	Guangdong	Meizhou	24.20293014	116.0798667
64	Guangdong	Shaoguan	24.81843298	113.7726202

65	Guangxi	Hechi	24.64377452	107.8398216
66	Guangxi	Liuzhou	24.94581318	109.3690902
67	Guangxi	Nanning	23.05811763	108.4652859
68	Guangxi	Qinzhou	22.1874002	109.0289448
69	Guizhou	Liupanshui	26.13595429	104.8890978
70	Guizhou	Zunyi	28.16859749	107.0868008
71	Hubei	Enshi	30.19249008	109.5498173
72	Hubei	Huanggang	30.71717017	115.3397949
73	Hubei	Wuhan	30.62649076	114.3441349
74	Hubei	Yichang	30.74985171	111.1377138
75	Hunan	Changde	29.29926483	111.5232718
76	Hunan	Changsha	28.22887147	113.1524111
77	Hunan	Chenzhou	25.81333594	113.1360848
78	Hunan	Huaihua	27.54796973	110.0743627
79	Hunan	Xiangtan	27.73394236	112.6031085
80	Hunan	Zhangjiajie	29.39282547	110.528969
81	Hunan	Zhuzhou	27.1206053	113.5176358
82	Jiangsu	Changzhou	31.63012132	119.634944
83	Jiangsu	Suzhou	31.37588841	120.6419371
84	Jiangsu	Taizhou	32.57267207	120.0561648
85	Jiangsu	Wuxi	31.52685876	120.0770537
86	Jiangsu	Yangzhou	32.73928264	119.4728542

87	Jiangxi	Ganzhou	25.70827198	115.2724875
88	Jiangxi	Xinyu	27.84797879	114.8520244
89	Shanghai	Shanghai	31.18546101	121.4208458
90	Sichuan	Guangyuan	32.26211873	105.7797223
91	Sichuan	Panzhihua	26.80752596	101.7312873
92	Sichuan	Zigong	29.28740643	104.6827647
93	Yunnan	Baoshan	24.97603196	98.97450111
94	Yunnan	Yuxi	24.13864646	102.1997581
95	Zhejiang	Jinhua	29.11688048	119.954105
96	Zhejiang	Shaoxing	29.7163403	120.6319989

Table S2. The list of model parameters.

Parameter	Range	Caveats
Mortality count	1 to 136	Dependent variable
Years of life lost	2.40 to 1527.60	Dependent variable
$PM_{2.5}$ (μg/m^3)	3.63 to 985.18	
Long-term trend	1 to 1461	Degrees of freedom = 6 per year
Day of the week	1 to 7	
Public holiday	0 and 1	
Temperature (°C)	-26.4 to 36.5	Degrees of freedom = 6
Relative humidity (%)	5.0 to 100.0	Degrees of freedom = 3

Abbreviations: $PM_{2.5}$ = particulate matter with an aerodynamic diameter less than or equal to 2.5 μm.

Table S3. Correlation coefficients between different air pollutants and meteorological conditions in the study cities.

	$PM_{2.5}$	SO_2	NO_2	O_3	Mean temperature	Relative humidity
$PM_{2.5}$	1					
SO_2	0.29	1				
NO_2	0.49	0.40	1			
O_3	0.36	0.36	-0.08	1		
Mean temperature	-0.15	-0.33	-0.28	0.20	1	
Relative humidity	-0.01	-0.14	-0.07	-0.12	0.24	1

Abbreviations: $PM_{2.5}$=particulate matter with an aerodynamic diameter less than or equal to 2.5 μm; SO_2=sulfur dioxide; NO_2=nitrogen dioxide; O_3=ozone.

Table S4. Regional-specific estimates of excess mortality risk and absolute change in years of life lost associated each 10 μg/m^3 increment in $PM_{2.5}$ in two-pollutant models.

Pollutants and Models	Excess mortality risk (95% CI)	YLL (95% CI)
Northwest		
Single-pollutant model	0.06 (-0.16, 0.29)	0.29 (-0.03, 0.61)
+ SO_2	0.06 (-0.17, 0.29)	0.30 (-0.00, 0.60)
+ NO_2	0.03 (-0.21, 0.26)	0.27 (-0.05, 0.59)
+ O_3	0.06 (-0.18, 0.29)	0.29 (-0.06, 0.65)
North		
Single-pollutant model	0.25 (-0.24, 0.74)	0.20 (-0.10, 0.50)
+ SO_2	0.17 (-0.41, 0.76)	0.12 (-0.23, 0.48)
+ NO_2	0.10 (-0.22, 0.42)	0.15 (-0.13, 0.43)
+ O_3	0.19 (-0.30, 0.68)	0.21 (-0.12, 0.53)
Northeast		
Single-pollutant model	0.08 (-0.04, 0.20)	0.18 (-0.12, 0.48)
+ SO_2	0.04 (-0.08, 0.17)	0.16 (-0.16, 0.49)
+ NO_2	0.05 (-0.08, 0.18)	0.04 (-0.28, 0.35)
+ O_3	0.09 (-0.08, 0.26)	0.16 (-0.16, 0.48)
Central		
Single-pollutant model	0.28 (-0.04, 0.60)	0.16 (-0.07, 0.39)
+ SO_2	0.19 (-0.09, 0.48)	0.14 (-0.09, 0.38)
+ NO_2	0.19 (-0.05, 0.44)	0.13 (-0.11, 0.37)
+ O_3	0.25 (-0.07, 0.57)	0.09 (-0.10, 0.29)
East		
Single-pollutant model	0.14 (0.04, 0.24)	0.23 (0.03, 0.43)
+ SO_2	0.16 (0.06, 0.27)	0.27 (0.06, 0.48)
+ NO_2	0.08 (-0.02, 0.18)	0.22 (0.01, 0.43)

+ O_3	0.14 (0.03, 0.25)	0.25 (0.05, 0.45)
Southwest		
Single-pollutant model	0.42 (-0.04, 0.89)	1.28 (0.14, 2.43)
+ SO_2	0.53 (0.10, 0.96)	1.58 (0.22, 2.95)
+ NO_2	0.38 (-0.03, 0.80)	1.35 (0.24, 2.47)
+ O_3	0.51 (0.16, 0.86)	1.31 (0.14, 2.48)
South		
Single-pollutant model	0.66 (0.38, 0.95)	0.74 (0.44, 1.03)
+ SO_2	0.75 (0.40, 1.09)	0.74 (0.39, 1.09)
+ NO_2	0.54 (0.23, 0.85)	0.74 (0.43, 1.04)
+ O_3	0.73 (0.42, 1.04)	0.85 (0.51, 1.18)
National effect estimates (pooled estimate)		
Single-pollutant model	0.22 (0.12, 0.31)	0.31 (0.19, 0.44)
+ SO_2	0.19 (0.10, 0.29)	0.31 (0.18, 0.43)
+ NO_2	0.14 (0.06, 0.22)	0.28 (0.16, 0.41)
+ O_3	0.20 (0.11, 0.30)	0.32 (0.19, 0.44)

Moving average concentration of lag 0 to lag 3 (lag_{03}) of daily $PM_{2.5}$ was used;

Abbreviations: $PM_{2.5}$=particulate matter with an aerodynamic diameter less than or equal to 2.5 μm; SO_2=sulfur dioxide; NO_2=nitrogen dioxide; O_3=ozone; YLL=years of life lost; CI, confidence interval.

Table S5. The absolute change in years of life lost from stroke per 10 μg/m^3 increment in $PM_{2.5}$ in mix-effect models.

Regions	Model 1	Model 2
Northwest	0.29 (-0.03, 0.61)	-0.89 (-1.17, -0.60)
North	0.20 (-0.10, 0.50)	-0.42 (-0.64, -0.19)
Northeast	0.18 (-0.12, 0.48)	0.45 (0.16, 0.74)
Central	0.16 (-0.07, 0.39)	0.46 (0.25, 0.67)
East	0.23 (0.03, 0.43)	0.23 (0.08, 0.37)
Southwest	1.28 (0.14, 2.43)	1.03 (0.57, 1.49)
South	0.74 (0.44, 1.03)	0.67 (0.41, 0.93)
National	0.31 (0.19, 0.44)	0.18 (0.09, 0.27)

Model 1: Three-stage model which generating the regional and national estimates by meta-analysis;

Model 2: Mix-effects regression model adjusting for the variable of city with a random term.

Table S6. Regional-specific estimates of excess mortality risk and absolute change in years of life lost associated each 10 μg/m³ increment of $PM_{2.5}$ in population-weighted model.

Regions	Excess mortality risk (95% CI)	YLL (95% CI)
Northwest	0.15 (-0.02, 0.32)	0.18 (-0.78, 1.13)
North	0.20 (-0.38, 0.79)	0.65 (0.08, 1.22)
Northeast	0.11 (-0.09, 0.31)	0.03 (-0.24, 0.29)
Central	0.33 (-0.22, 0.88)	0.66 (-0.26, 1.58)
East	0.29 (0.12, 0.45)	0.74 (0.35, 1.13)
Southwest	0.36 (-0.52, 1.25)	3.35 (-1.93, 8.64)
South	1.03 (0.56, 1.50)	1.12 (0.51, 1.72)
National	0.32 (0.15, 0.49)	0.95 (0.33, 1.57)

Abbreviations: $PM_{2.5}$=particulate matter with an aerodynamic diameter less than or equal to 2.5 μm; YLL=years of life lost; CI, confidence interval.

Table S7. The avoidable stroke-related years of life lost, potential gains in life expectancy and PAF for those dying of ischemic stroke by enhancing $PM_{2.5}$ level to Chinese and WHO's guidelines in the study cities during 2013-2016.

Region	Avoidable YLL (95% CI)		Benefits in life expectancy (95% CI)		PAF (%, 95% CI)	
	China's standard (IT-1)	WHO's AQG	China's standard (IT-1)	WHO's AGQ	China's standard (IT-1)	WHO's AQG
Northwest	-17.28 (-78.74, 44.19)	-59.45 (-767.96, 649.05)	0.01 (-0.05, 0.07)	-0.09 (-0.59, 0.42)	0.06 (-0.44, 0.57)	-0.65 (-4.96, 3.66)
North	106.18 (-124.47, 336.84)	415.14 (-383.07, 1213.34)	0.01 (-0.01, 0.03)	0.08 (-0.04, 0.19)	0.11 (-0.08, 0.30)	0.73 (-0.39, 1.84)
Northeast	85.49 (-45.69, 216.68)	479.41 (-340.14, 1298.95)	0.02 (-0.01, 0.04)	0.08 (-0.04, 0.19)	0.13 (-0.06, 0.33)	0.60 (-0.37, 1.56)
Central	-0.11 (-43.47, 43.26)	129.95 (-341.59, 601.50)	0.01 (-0.02, 0.03)	0.12 (-0.01, 0.24)	0.10 (-0.17, 0.38)	1.15 (-0.06, 2.35)

East	234.67 (81.02, 388.31)	1113.60 (483.62, 1743.58)	0.04 (0.02, 0.06)	0.18 (0.10, 0.27)	0.43 (0.20, 0.67)	1.95 (1.12, 2.79)
Southwest	3.41 (-0.24, 7.06)	603.77 (37.26, 1170.27)	0.001 (-0.0001, 0.002)	0.31 (0.19, 0.44)	0.01 (-0.001, 0.02)	3.02 (1.81, 4.23)
South	12.75 (-21.14, 46.64)	464.47 (24.19, 904.75)	0.02 (-0.01, 0.06)	0.14 (0.03, 0.24)	0.24 (-0.08, 0.56)	1.33 (0.30, 2.36)
National	4.19 (0.91, 7.46)	507.45 (263.73, 751.17)	0.03 (0.01, 0.04)	0.13 (0.08, 0.18)	0.24 (0.12, 0.35)	1.21 (0.73, 1.70)

Moving average of lag 0 to lag 3 (lag_{03}) for daily $PM_{2.5}$ level was used;

The reference $PM_{2.5}$ standards were the IT-1 or Chinese NAAQS (75 μg/m^3) and WHO's AQG (25 μg/m^3);

Abbreviations: $PM_{2.5}$ = particulate matter with an aerodynamic diameter less than or equal to 2.5 μm; YLL = years of life lost; PAF = population attributable fraction; IT = interim targets; NAAQS = National Ambient Air Quality Standards; AQG = Ambient Air Quality Guidelines.

Table S8. The avoidable stroke-related years of life lost, potential gains in life expectancy and PAF for those dying of hemorrhagic stroke by enhancing $PM_{2.5}$ level to Chinese and WHO's guidelines in the study cities during 2013-2016.

Region	Avoidable YLL (95% CI)		Benefits in life expectancy (95% CI)		PAF (%, 95% CI)	
	China's standard (IT-1)	WHO's AQG	China's standard (IT-1)	WHO's AGQ	China's standard (IT-1)	WHO's AQG
Northwest	25.20 (-1.09, 51.49)	427.45 (-172.09, 1026.99)	0.02 (-0.02, 0.06)	0.16 (-0.03, 0.34)	0.13 (-0.14, 0.39)	1.07 (-0.18, 2.32)
North	-9.56 (-62.15, 43.02)	32.07 (-666.15, 730.30)	0.003 (-0.03, 0.04)	0.07 (-0.11, 0.25)	0.01 (-0.20, 0.22)	0.44 (-0.72, 1.59)
Northeast	29.49 (-134.10, 193.08)	198.65 (-721.59, 1118.89)	0.02 (-0.01, 0.04)	0.06 (-0.04, 0.16)	0.10 (-0.04, 0.23)	0.38 (-0.19, 0.96)
Central	5.66 (-91.51, 102.83)	78.08 (-694.96, 851.13)	0.004 (-0.02, 0.03)	0.05 (-0.09, 0.19)	0.04 (-0.16, 0.24)	0.35 (-0.64, 1.34)

East	2.65 (-13.01, 18.31)	-73.93 (-494.54, 346.67)	0.005 (-0.01, 0.02)	0.002 (-0.08, 0.08)	0.04 (-0.03, 0.11)	0.06 (-0.55, 0.66)
Southwest	0.61 (-3.37, 4.59)	534.04 (-26.03, 1094.11)	0.0001 (-0.001, 0.001)	0.13 (-0.03, 0.29)	0.001 (-0.01, 0.01)	1.02 (-0.17, 2.21)
South	38.99 (-23.04, 101.03)	796.52 (108.37, 1484.67)	0.02 (-0.01, 0.04)	0.12 (0.001, 0.24)	0.13 (-0.05, 0.31)	0.89 (0.04, 1.75)
National	2.58 (-2.31, 7.47)	266.17 (18.67, 513.67)	0.0001 (-0.0006, 0.0009)	0.07 (0.02, 0.12)	0.001 (-0.004, 0.01)	0.50 (0.18, 0.82)

Moving average of lag 0 to lag 3 (lag_{03}) for daily $PM_{2.5}$ level was used;

The reference $PM_{2.5}$ standards were the IT-1 or Chinese NAAQS (75 μg/m^3) and WHO's AQG (25 μg/m^3);

Abbreviations: $PM_{2.5}$ = particulate matter with an aerodynamic diameter less than or equal to 2.5 μm; YLL = years of life lost; PAF = population attributable fraction; IT = interim targets; NAAQS = National Ambient Air Quality Standards; AQG = Ambient Air Quality Guidelines.

Table S9. Change in the association between $PM_{2.5}$ and YLL for each IQR increment in city characteristics.

City-level variables	IQR	Change in $PM_{2.5}$–YLL relationship (%)	*P*-value
Gross Domestic Product (in billions, CNY)	218.20	0.18 (0.06, 0.30)	<0.001
Population density (person/square kilometer)	1874.04	-0.03 (-0.09, 0.04)	0.42
GDP per capita (in thousands, CNY)	29.36	0.01 (-0.04, 0.06)	0.77
Elevation (m)	836.48	-0.33 (-0.88, 0.22)	0.24
Precipitation (mm)	58.53	-0.31 (-0.84, 0.22)	0.25
Poverty rate (%)	24.16	0.06 (-0.22, 0.34)	0.65
Education level (by year of school)	1.78	-0.21 (-0.55, 0.13)	0.23
Annual $PM_{2.5}$ concentration (μg/m^3)	36.77	-0.17 (-0.64, 0.3)	0.48
Annual O_3 concentration (μg/m^3)	20.45	-0.26 (-0.51, -0.01)	0.04
Annual SO_2 concentration (μg/m^3)	24.52	0.31 (-0.01, 0.62)	0.06
Annual NO_2 concentration (μg/m^3)	14.45	-0.33 (-0.74, 0.09)	0.12
Annual air pressure (kPa)	60.83	-0.20 (-0.64, 0.24)	0.38
Temperature (℃)	6.15	0.39 (0.01, 0.77)	0.04
Relative humidity (%)	18.85	-0.12 (-0.71, 0.46)	0.68

Abbreviations: $PM_{2.5}$ = particulate matter with an aerodynamic diameter less than or equal to 2.5 μm; YLL = years of life lost; IQR = the difference between the third quartile and the first

quartile; CNY = Chinese Yuan; CO = carbon monoxide; O_3 = ozone; SO_2 = sulfur dioxide; NO_2 = nitrogen dioxide.

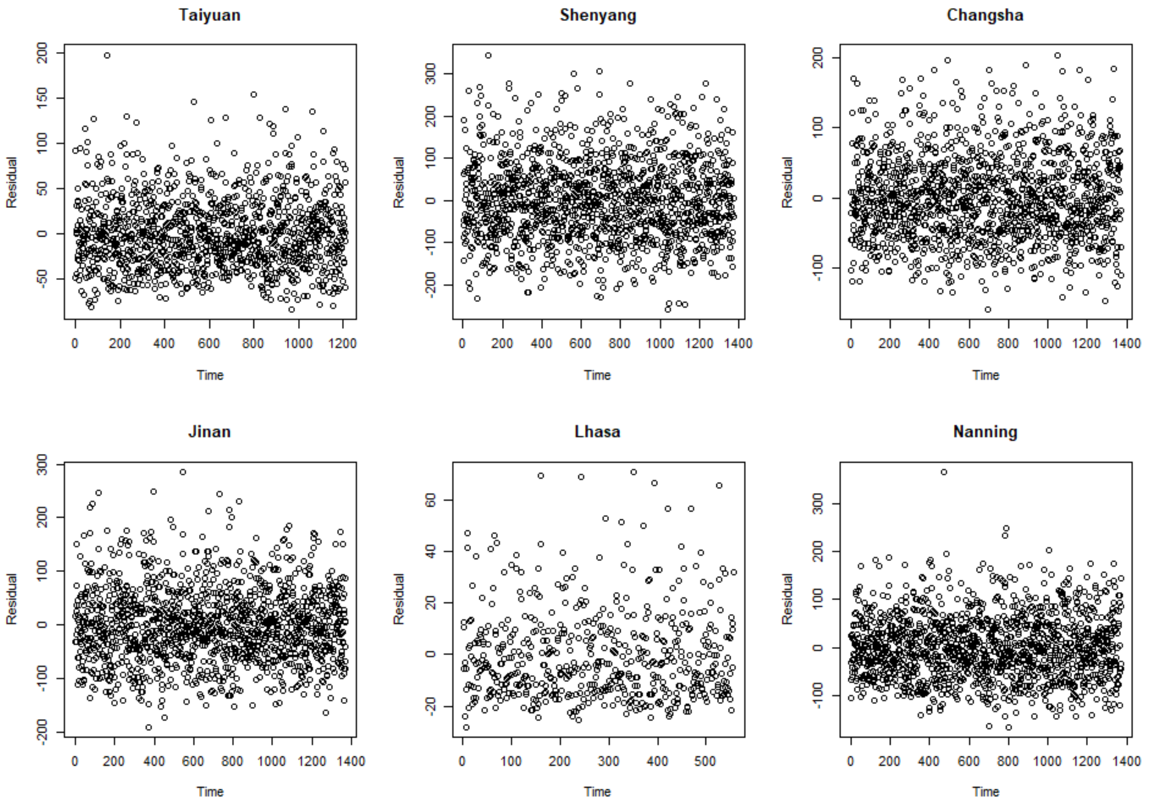

Figure S1. The residual plot for six provincial capitals.

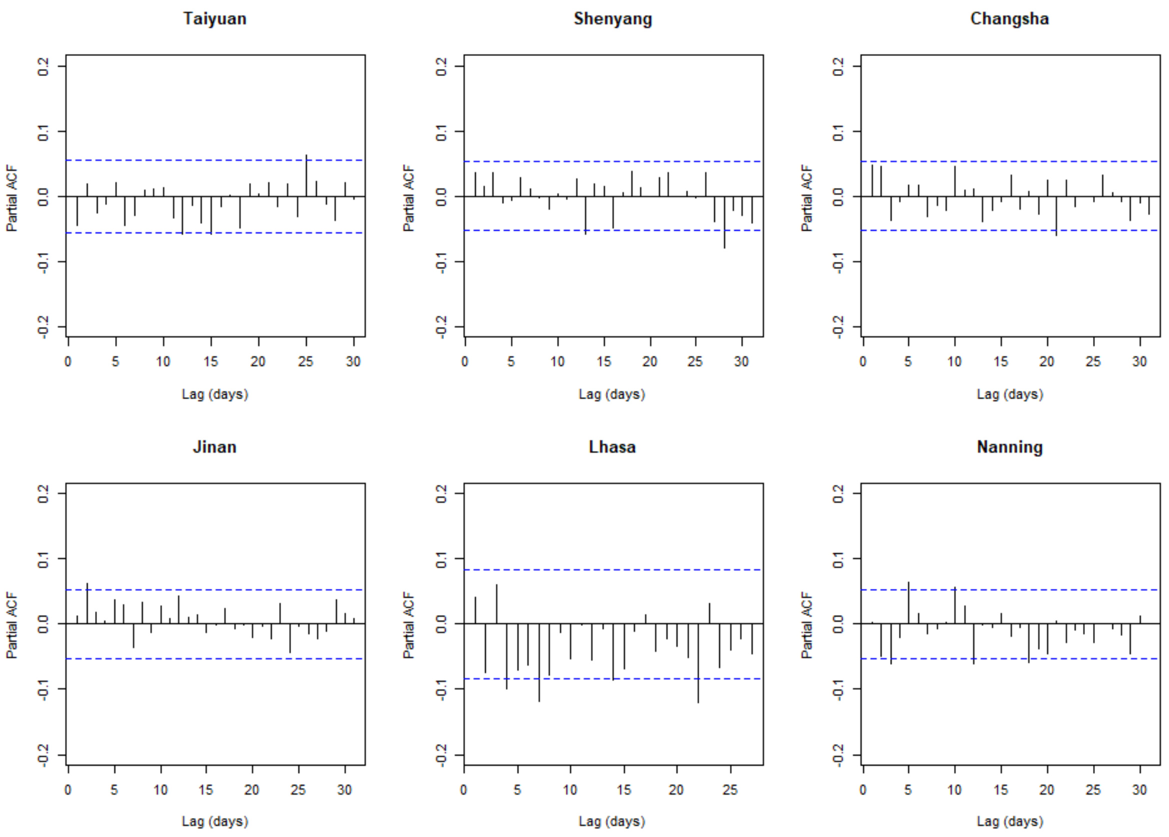

Figure S2. The partial ACF plot for six provincial capitals. Abbreviations: ACF = autocorrelation function.

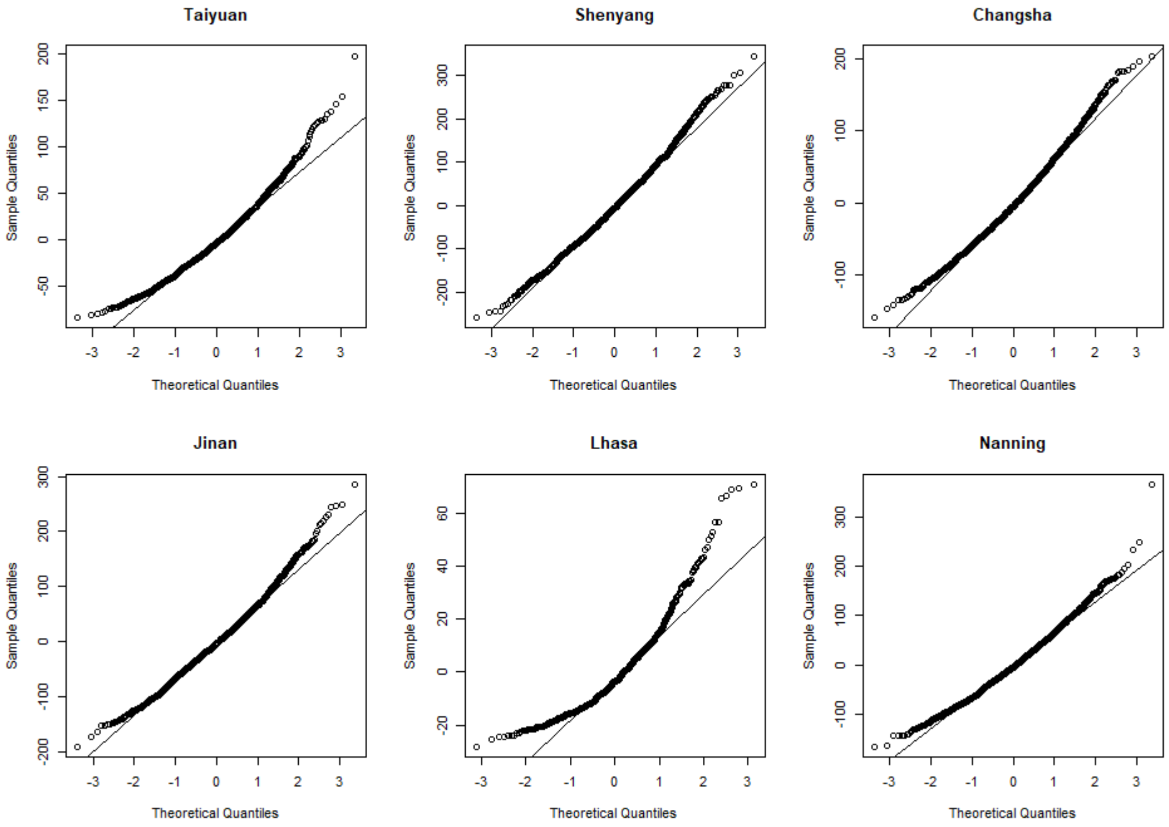

Figure S3. The Q-Q plots for six provincial capitals.
